# The influence of the extraction method on bioactivity of the root of *Tetrastigma hemsleyanum*

**DOI:** 10.1002/fsn3.1221

**Published:** 2019-10-21

**Authors:** Yangyang Liu, Xiang Ye, Yonglu Li, Qiang Chu, Lushuang Yu, Wen Chen, Ruoyi Jia, Yong Jiang, Xiaodong Zheng

**Affiliations:** ^1^ Department of Food Science and Nutrition Zhejiang University Hangzhou China; ^2^ Zhejiang Key Laboratory for Agro‐food Processing Zhejiang University Hangzhou China; ^3^ Fuli Institute of Food Science Zhejiang University Hangzhou China; ^4^ Shanghai Zhengyue Enterprise Management Co., Ltd. Shanghai China

**Keywords:** anti‐inflammatory, antioxidant activity, antiproliferative, extraction, *Tetrastigma hemsleyanum*

## Abstract

*Tetrastigma hemsleyanum* is traditionally used as a folk medicine and functional food in China. Its extracts have been confirmed to have many bioactivities. However, the effect of extracting temperature on its bioactivity has not been reported. In this research, the total flavonoids content (TFC), total polyphenol content (TPC), antiproliferative, antioxidant, and anti‐inflammatory activities of ethanol extracts and water extracts (extracted at 55, 70, 85, and 100°C) were observed. The results indicated that ethanol extracts showed better antioxidant activity with DPPH EC_50_ of 504.1 ± 3.8 μg/ml and ABTS EC_50_ of 851.4 ± 3.9 μg/ml. A better antiproliferative activity on HepG2, PC12, Caco‐2, and Hela cells was observed on ethanol extracts. The results of anti‐inflammatory activities also indicated that all of the extracts can reduce the NO production of LPS‐stimulated macrophage with dose‐independent manner. In summary, the results showed that the antiproliferative, antioxidant, and anti‐inflammatory activities of water extracts decreased with the increasing temperature to some extent.

## 1 INTRODUCTION

As many natural products are confirmed to have many bioactivities, such as antioxidant and anti‐aging activities, the extracts of natural products captured many researchers' attention (Chen, Zhang, Chen, Han, & Gao, 2017). However, the composition and bioactivities of extracts of natural products were usually affected by the extracting method, solvent, and temperature (Cvetanović et al., 2019; Gayathri et al., 2019; Pimentel‐moral et al., 2018; Sun et al., 2016; Wang, Liu, & Hu, 2014; Yu, Ji, Yang, & Liu, 2019; Zhang et al., 2016). In view of this, the extraction temperature and solvent are necessary factors should be taken into consideration when the bioactivity evaluation of a natural product was performed.

*Tetrastigma hemsleyanum* Diels et Gilg belongs to the genus *Tetrastigma* Planch, family Vitaceae. It is distributed in southern China, such as Zhejiang Province, Jiangxi Province, Hunan Province, Fujian Province, and Guangxi Province. Its root and whole grass have many bioactivities which can be used as herbal medicine (Sun et al., 2018, 2013, 2017). It is especially famous for its root which is widely used for removing pathogenic heat from blood, activating blood, and alleviating pain. Modern pharmacological studies show that *T. hemsleyanum* has a wide range of pharmacological functions, such as antiproliferative, antivirus, anti‐inflammatory, antidiabetic, liver protective, and immunoregulation activities (Ding et al., 2008; Ma et al., 2012; Ru et al., 2019; Wang et al., 2018, 2016; Xu et al., 2008; Yang et al., 1989; Yang & Wu, 2009; Zhong, Mao, Huang, & Wei, 2006). It is known as a plant antibiotic with the Chinese name “sanyeqing.” Among these bioactivities, antitumor activity is considered to be the most important function. Studies showed that the root of *T. hemsleyanum* could inhibit cutaneous squamous carcinoma A431 cell line (Zhong, Yang, Zhu, Peng, & Cao, 2019), liver carcinoma HepG2 cell line (Peng, Zhuang, & Guo, 2015; Sun et al., 2018), lung carcinoma A549 cell line (Zeng et al., 2012), human cervical carcinoma HeLa cell line (Xiong, Wu, & Rao, 2015), and breast carcinoma MDA‐MB‐435S cell line (Lin, Chen, Qiu, & Guo, 2016).

In previous study, many compounds were detected and identified in the extracts of the root of *T. hemsleyanum*, such as (a) β‐sitosterol (Xiong et al., 2015), (b) palmitic acid, (c) protocatechuic acid, (d) salicylic acid, (e) p‐hydroxybenzoic acid, (f) resveratrol, (g) *trans*‐4‐hydroxycinnamic acid, (h) kaempferol, (i) quercetin, (j) isoquercitrin (Lin et al., 2016; Xia, Li, & Zhou, 2018), (k) vitexin, (l) isovitexin (Fan et al., 2016), (m) rutin (Sun et al., 2015), (n) catechin, (o) kaempferol‐3‐robinoside‐7‐rhamnoside, 5‐caffeoylquinic acid, (p) kaempferol‐3‐rutinoside, (q) kaempferide, (r) orientin (Sun et al., 2017). What's more, many of these compounds have been reported to have bioactivity.

Though many studies have been done on the compounds and bioactivities of extracts of *T. hemsleyanum*, the effects of extracting temperature of extracts of *T. hemsleyanum* on its bioactivities have not been reported. In this research, we aimed to investigate the effect of extraction solvent and temperature in the extracting process of *T. hemsleyanum* on the viability of human cancer cell line (HepG2, PC‐12, Hela, Caco‐2) and response of RAW264.7 macrophage to LPS as an in vitro inflammation model. Also, antioxidant activity of *T. hemsleyanum* was observed with DPPH, ABTS, and FRAP assays.

## 2 EXPERIMENTAL

### 2.1 Materials

*Tetrastigma hemsleyanum w*ere obtained from Lishui Zhejiang China. Carbon dioxide with 99.9 mol% was supplied by Jingong Gas Co., Ltd. Methanol (GC grade) was purchased from Sigma. Acetonitrile (GC grade) was purchased from Aladin. 2,2′‐diphenyl‐1‐picrylhydrazyl (DPPH), vitamin C (VC), and 2,4,6‐tri(2‐pyridyl)‐1,3,5‐triazine (TPTZ) were purchased from Aladin, and all the other reagents used for analysis were of analytical grade. Water was purified by using a Milli‐Q system from Millipore.

### 2.2 Extraction procedures

The root of *T. hemsleyanum* was ground into powder and strain through a 60‐mesh sieve. The powder was extracted with water or ethanol with the ratio of 1:10 (w: v) at different temperature with ultrasonic treatment. Approximately 100.00 g of the powder samples was accurately weighed and loaded into a beaker with 1L water, which was placed in the water bath or ultrasonic equipped with a temperature controller (±0.1°C). The suspension was stirred for 1.5 hr. This mixture was filtered through a qualitative filter, and the residue was added to 1 L water for extracting again. The extraction and filtration were repeated for three times, and the filtrate was combined and centrifuged at 4,000 g for 10 min at 4°C. The supernatant was concentrated with a spin evaporator to nearly 100 ml, then the concentration was centrifuged at 8,000 g for 10 min at 4°C, and the supernatant was lyophilized and stored in −80°C until use.

The water extraction was carried out at 55, 70, 85, and 100°C, and the extracts were described as THW55, THW70, THW85, and THW100, respectively. The ultrasonic extraction was carried out at 55°C with solvent of water and 80% ethanol, and the extracts were described as THWU and THEU, respectively. Though the cavitation at 55°C is not the best choice for ethanol. To compare the bioactivities of water extracts extracted at 55°C, we selected 55°C as ultrasound extraction for ethanol. Each experiment was repeated three times to assure statistically valid results.

### 2.3 Yield of extraction calculation

The extraction yield was obtained with the following equation:

$$\text{Yield}=\frac{\text{weight}\;\text{of}\;\text{lyophilized}\;\;\text{extracts}\;(\text{mg})}{\text{weight}\;\text{of}\;\text{initial}\;Tetrastigma\;hemsleyanum\;\text{root}\;\text{sample}\;(g)}$$

### 2.4 Total flavonoids content

Total flavonoids content assay was according to Zheng (Zheng et al., 2018) with small modification. Briefly, 500 μl samples were mixed with 30 μl 0.75 M NaNO_2_ to react for 5 min at room temperature. Then, 30 μl 0.4 M Al(NO_3_)_3_ was added and allowed to react for 6 min at room temperature. Then, 400 μl 1 M NaOH was added. Subsequently, the absorbance of the sample was measured at 510 nm with a UV spectrophotometer. TFC was expressed as rutin equivalent per gram lyophilized weight sample (mg rutin/ g DW) by using the rutin calibration curve.

### 2.5 Total polyphenol content

The polyphenol of extracts of the root of *T. hemsleyanum* was determined according to Sun (Sun et al., 2013) with small modification. Briefly, 100 μl samples were mixed with 200 μl 10% Foline‐Phenol. Then, 800 μl 700 mM Na_2_CO_3_ was added and allowed to react for 120 min at room temperature in the dark. Subsequently, the absorbance of the sample was measured at 765 nm with a UV spectrophotometer. TPC was expressed as gallic acid equivalent per gram lyophilized weight sample (mg gallic acid/ g DW) by using the gallic acid calibration curve.

### 2.6 HPLC analysis

The extracts were analyzed using HPLC (Thermo Fisher Scientific) equipped with a DAD detector. The extracts were dissolved in deionized water at a concentration of 5 mg/ml, and the solutions were filtered with 0.22 μm membrane. Then, the samples were injected into the HPLC system to be analyzed. The total analysis time was 60 min.

The mobile phase consists of acetonitrile (A) and 0.1% formic acid (B), which was pumped into HPLC system at 0.8 ml/min with the following gradient elution program: 0–5 min, 5%A; 5–25 min, 5%–16%A; 25–33 min, 16%–30%A; 33–35 min, 30%–90%A; 35–40 min, 90%A; 40–45 min, 90%–5%A; 45–50 min, 5%A. The absorbance was detected at 360 nm.

### 2.7 Antioxidant activity evaluation

Three methods were used to assess the antioxidant activity of extracts: reduction of the 2,2‐diphenyl‐1‐picrylhydrazyl radical (DPPH), reduction power of the ferric ion (FRAP), and reduction of the 2,2‐azino‐bis(3‐ethylbenzothiazoline‐6‐sulfonate) cation (ABTS). The assays were according to the method of Gonçalves (Gonçalves et al., 2019) with small modification. 50% of antioxidant activity was described as EC50 which was obtained from the graphs of antioxidant activity versus sample concentrations.

### 2.8 DPPH scavenging capacity test

Radical scavenging activities of extracts were determined according to DPPH scavenging capacity assay.

20 μl samples (100–1,000 μg/ml) were mixed with 700 μl of 0.1 mM DPPH in ethanol. The mixture was incubated at 37°C in the dark for 30 min. Absorbance of the sample was measured using a UV–visible spectrophotometer at 517 nm against water as blank. The percent of DPPH radical inhibition of the samples was calculated according to the following equation:

$$\;\text{percentage}\;\text{of}\;\text{DPPH}\;\text{radical}\;\text{inhibition}\;\left(\%\right)=\frac{A_{i}\;-A_{0}}{A_{0}}\times 100\%$$

where *A_i_* represents the absorbance of samples and *A_0_* denotes the absorbance of the blank sample. All samples were tested in triplicate.

### 2.9 ABTS assay

20 μl samples at different concentrations (100–1,000 μg/ml) were allowed to react with 700 μl fresh ABTS solution for 6 min at room temperature in the dark. The absorbance of the sample was measured at 734 nm with a UV spectrophotometer. The percent of DPPH radical inhibition of the samples was calculated according to the following equation:

$$\;\text{percentage}\;\text{of}\;\text{ABTS}\;\text{radical}\;\text{inhibition}\;\left(\%\right)=\frac{A_{i}\;-A_{0}}{A_{0}}\times 100\%$$

where *A_i_* represents the absorbance of samples and *A_0_* denotes the absorbance of the blank sample. All samples were tested in triplicate.

### 2.10 FRAP assay

A sample (100–1,000 μg/ml) with the volume of 20 μl was mixed with 700 μl ferric‐TPTZ reagent (mixed by 10 mM TPTZ in 40 mM HCl, 20 mM FeCl_3_.6H_2_O, 300 mM acetate buffer solution, PH 3.5, at a ratio of 1:1:10 [v: v: v]). The absorbance of the sample was measured at 734 nm with a UV spectrophotometer. The antioxidant activities were expressed as VC equivalent per gram dry weight sample. All samples were tested in triplicate.

### 2.11 Antiproliferative activity of extracts on cancer cells

Human liver carcinoma HepG2 cells, human cervical carcinoma Hela cells, human colon carcinoma Caco‐2 cells, and adrenal pheochromocytoma PC12 cells were all purchased from the Cell Bank of Type Culture Collection of Chinese Academy of Sciences. The cell was grown in complete DMEM supplemented with 10% of fetal bovine serum (v/v), 100 IU/ml penicillin, and 100 μg/ml streptomycin at 37°C in a humidified incubator including 5% CO_2_ atmosphere.

To compare the effect of extracts of *T. hemsleyanum* on cancer cells, MTT assays were performed with HepG2 cells. The cells were washed with PBS followed by 24‐hr incubation with the extracts and incubated for 4 hr with MTT solution with a final concentration of 0.5 mg/ml. The purple formazan crystals produced by viable metabolically active cells were dissolved by DMSO. The absorbance of the sample was measured at 570 nm with a UV spectrophotometer. Cell viability was compared by IC_50_ values.

### 2.12 Anti‐inflammatory activity

#### 2.12.1 Cell culture

The mouse macrophage cell line RAW 264.7 was provided by the Cell Bank of Type Culture Collection of Chinese Academy of Sciences. The cell was grown in complete DMEM supplemented with 10% of fetal bovine serum (v/v), 100 IU/ml penicillin, and 100 μg/ml streptomycin at 37°C in a humidified incubator including 5% CO_2_ atmosphere.

#### 2.12.2 MTT assays

MTT assay was used to investigate the effect of extracts of *T. hemsleyanum* on RAW 264.7 cells*.* Briefly, 6* × *10^3^ RAW 264.7 cells/well were seeded into a 96‐well plate. After incubated for 24 hr, cells were treated with extracts at different concentrations (200, 400, 600, and 800 μg/ml). After another 24 hr, the cells were washed with PBS and incubated with MTT (0.5 mg/ml) for 4 hr, and the plate was centrifuged at 2,800 g. Then, the MTT was removed and 150 μl dimethyl sulfoxide was added to dissolve the formazan precipitate. The absorbance of the sample was measured at 570 nm with a UV spectrophotometer.

#### 2.12.3 Nitric oxide (NO) quantification in macrophage cell culture medium

RAW 264.7 cells were seeded in a 6‐well plate and incubated for 12 hr to make the cells adhere. Cells were pretreated with extracts at different concentrations (200, 400, 600, and 800 μg/ml) for 12 hr. Then, the medium was removed and the cells were cotreated with the same extract concentration medium containing 1 μg/ml of LPS for 24 hr. Finally, the medium was collected and nitric oxide (NO) was tested by Griess reagent.

Nitrite accumulation, and indicator of synthesis, was measured in the medium that RAW 264.7 cultured according to the total nitric oxide assay kit instructions manufactured by Beyotime. Briefly, 50 μl of supernatant was reacted with reagent I; thereafter, 50 μl reagent II was added, the plate was incubated for 5 min, and the absorbance of the sample was measured at 550 nm with a UV spectrophotometer. A sodium nitrite standard curve was used to accumulate the amount of NO. All samples were tested in triplicate.

### 2.13 Statistical analysis

All of the experimental data were presented as mean ± deviation (*SD*). Statistical significance was evaluated by one‐way analysis of significance (ANOVA) followed by the Tukey test (SPSS 22.0 software, IBM Inc). *p* < .05 was regarded to be significantly different in statistics.

## 3 RESULTS AND DISCUSSION

### 3.1 The yield of extracts

The yields of extracts of *T. hemsleyanum* at different extracting methods are shown in Table 1. The yield considerably differed among different temperatures. THEU had the lowest yield (*p* < .05). However, THW100 had the highest yield. There is no significant difference between the yields of THWU and THW55.

**Table 1:** The yield, total flavonoids content (TFC), and total polyphenol content (TPC) of extracts of *Tetrastigma hemsleyanum* of different extraction methods

Extracts	Yield (mg/g sample)	TFC (mg rutin/ g DW)	TPC (mg gallic acid/g DW)
THEU	45.5 ± 5.4d	392.8 ± 5.0b	239.9 ± 3.2a
THWU	79.8 ± 3.2c	328.3 ± 4.6c	240.9 ± 3.5a
THW55	76.5 ± 6.4c	431.4 ± 9.5a	239.4 ± 4.3a
THW70	73.8 ± 8.1c	266.3 ± 9.3d	198.5 ± 5.3b
THW85	86.8 ± 1.8b	222.1 ± 4.1e	170.1 ± 1.5c
THW100	106.5 ± 4.9a	216.0 ± 4.0e	90.6 ± 2.9d

Note

The data represent the mean values ± standard Deviation of three independent experiments.

The difference among different group were compared at p <0.05 probability level the minuscule represent the difference among the same line (different dose of same extracts).

### 3.2 Quantification of TFC and TPC in extracts of *Tetrastigma hemsleyanum*

As shown in Table 1, different extraction methods affected the total flavonoids contents of the root of *T. hemsleyanum.* The TFC of extracts in ethanol extraction was similar to water extraction at 55°C but higher than water extraction at 70, 85, and 100°C (*p* < .05). At low temperature, the TFC of extracts of water extraction was higher than that at high temperature. To some extent, the TFC decreases with the increasing temperature. This phenomenon may account for that more polysaccharide was extracted at 85°C and 100°C than at 55°C.

Table 1 indicates that the ethanol extracts had similar TPC with water extracts extracted by ultrasonic. As for water extracts, the TPC decreased with the increasing temperature (*p* < .05).

### 3.3 Characterization of the chemical constituents of *Tetrastigma hemsleyanum*

**Figure 1:**
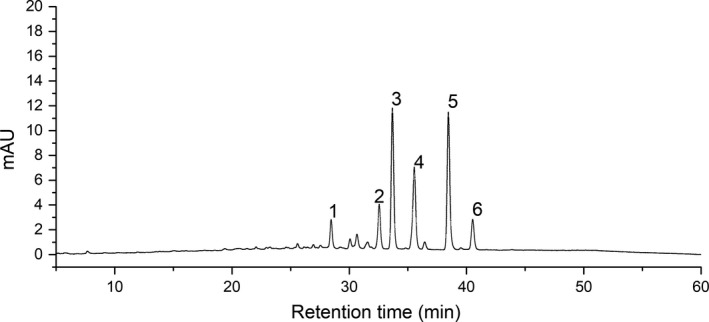
The base peak chromatograms of extracts of *Tetrastigma hemsleyanum* detected at wavelength of 360 nm

A list of identified major compounds in ethanol extracts of *T. hemsleyanum* by UPLC‐TOF‐MS is shown in Figure 1 and Table 2. Figure 1 shows the base peak chromatogram in the DAD at 360 nm. These peaks in Figure 1 were summarized with their retention times in Table 2. According to our previous study (Li et al., 2019), the major flavone compounds were proanthocyanidin dimer (a), kaempferol‐3‐xylosylglucose‐7‐rhamnoside (b), rutin (c), quercetin‐3‐O‐glucoside(isoquercitrin) (d), kaempferol‐3‐O‐rutinoside (e), and kaempferol‐3‐O‐glucoside(astragalin) (f). More data about other compounds and its MS data were in our previous study (Li et al., 2019). What's more, these compounds were also detected in other study about *T. hemsleyanum* (Fan et al., 2016; Lin et al., 2016; Sun et al., 2015, 2017; Xia et al., 2018). As Table 2 shows, the peak area differs in different extracts which indicated that major compound content differs in different extracts.

**Table 2:** The peak area of different extracts of *Tetrastigma hemsleyanum*

NO	Retention time (min)	Compounds	Formula	mAU*min					
				THEU	THWU	TH55	TH70	TH85	TH100
1	28.49	Proanthocyanidin dimer	C_30_H_26_O_12_	3.423 ± 0.211a	0.721 ± 0.052b	0.832 ± 0.007b	0.612 ± 0.005bc	0.600 ± 0.029bc	0.492 ± 0.027c
2	32.59	Kaempferol−3‐xylosylglucose−7‐rhamnoside	C_32_H_38_O_19_	6.258 ± 0.412a	1.308 ± 0.057b	1.651 ± 0.141b	1.117 ± 0.012bc	1.107 ± 0.035bc	0.859 ± 0.050c
3	33.72	Rutin	C_27_H_30_O_16_	6.157 ± 0.393a	3.757 ± 474c	4.653 ± 0.212b	3.290 ± 0.034c	3.167 ± 0.099c	2.703 ± 0.171c
4	35.60	Quercetin−3‐O‐glucoside	C_26_H_28_O_15_	4.964 ± 0.322a	2.474 ± 0.011c	3.124 ± 0.163b	2.068 ± 0.015c	2.192 ± 0.063c	1.864 ± 0.114c
5	38.50	Kaempferol−3‐O‐rutinoside	C_27_H_30_O_15_	5.946 ± 0.398a	3.870 ± 0.107c	4.844 ± 0.141b	3.434 ± 0.044c	3.265 ± 0.111c	2.790 ± 0.193d
6	40.57	Kaempferol−3‐O‐glucoside	C_21_H_27_O_11_	1.693 ± 0.094a	0.854 ± 0.059c	1.088 ± 0.025b	0.692 ± 0.008c	0.727 ± 0.015c	0.652 ± 0.038c

Note

* The difference among different group were compared at *p* < .05 probability level the minuscule represent the difference among the same row (different dose of same extracts).

### 3.4 Estimation of antioxidant activity of extracts of *Tetrastigma hemsleyanum* in vitro

**Figure 2:**
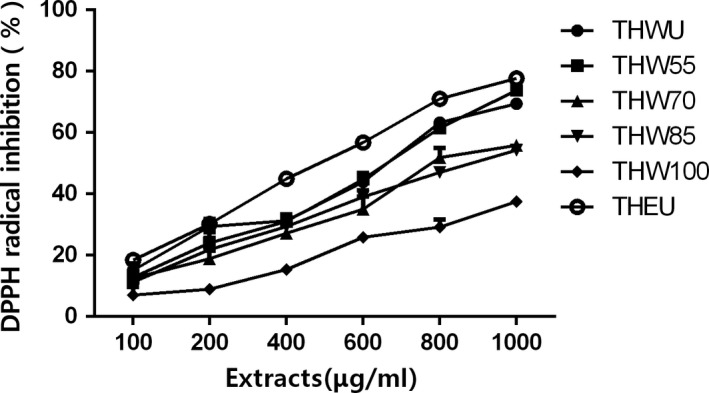
Scavenging DPPH capacity of *Tetrastigma hemsleyanum*

As Figure 2 shows, all of the extracts had the DPPH radical scavenging ability. The scavenging capacity of all the extracts increased with the increasing dose is observed in Figure. In other words, the scavenging capacity had positive correlation with dose. However, at the same dose, the scavenging ability differed among all the extracts. The EC_50_ of the DPPH radical scavenging ability is shown in Table 3. The percent of DPPH radical inhibition of THEU was higher than that of THWU and all the other water extracts (*p* < .05). As for water extracts, the percent of DPPH radical inhibition of THW55 was highest and that of THW100 was the lowest on the contrary. The percent of DPPH radical inhibition of THW70 and THW85 was similar. This phenomenon may account for the difference of TPC. Many studies showed that polyphenols had the ability to scavenge the free radical.

**Table 3:** In vitro antioxidant activity of extracts of different extracting methods

Extracts	DPPH EC_50_ (μg/ml)	ABTS EC_50_ (μg/ml)	FRAP (mg VC/g DW)
THEU	504.1 ± 3.8e	851.4 ± 3.9d	235.3 ± 18.9a
THWU	619.4 ± 22.4d	1,075.9 ± 22.8c	188.1 ± 14.2b
THW55	641.1 ± 22.4d	1,009.85 ± 10.0c	189.2 ± 14.1b
THW70	873.6 ± 27.3c	1,080.3 ± 11.6c	162.6 ± 19.3c
THW85	1,022.1 ± 30.6b	1,495.3 ± 19.8b	134.9 ± 10.8d
THW100	1562.4 ± 72.6a	1,820.4 ± 37.8a	114.4 ± 10.1e

Note

The data represent the mean values ± standard deviation of three independent experiments.

The difference among different group was compared at *p* < .05 probability level the minuscule represent the difference among the same line (different dose of same extracts).

As Figure 2 shows, all of the extracts had ABTS radical scavenging capacity. Among all the extracts, the percent of ABTS radical inhibition showed dose‐independent manner. THEU had the highest scavenging ability with the EC_50_ of 719.1 ± 10.4 μg/ml. As for water extracts, the EC_50_ of ABTS radical scavenging ability had the same trends with EC_50_ of DPPH radical scavenging. The EC_50_ of ABTS radical scavenging increased with extracting temperature. In other words, the water extracts obtained at low temperature had higher scavenging ability than that extracted at high temperature.

The results of ferric reducing antioxidant power (FRAP) assays are shown in Table 3. As expected, the THEU had the highest reducing antioxidant power with the value of 235.3 ± 18.9 mg VC/g DW (*p* < .05). As the extracting temperature increased, the VC equivalents in extracts decreased which indicated that the antioxidant capacity of water extracts had negative correlation with extracting temperature.

In summary, all of the three assays of DPPH, ABTS, and FRAP indicated that the ethanol extracts had the best antioxidant activity and antioxidant activity of water extracts in vitro decreased with increasing temperature (*p* < .05). This phenomenon could be due to the difference content of rutin and other flavones which have been considered to have antioxidant activity. Moreover, the antioxidant activity of all extracts showed dose‐independent manner to some extent.

### 3.5 The antiproliferative activity of extracts

**Figure 3:**
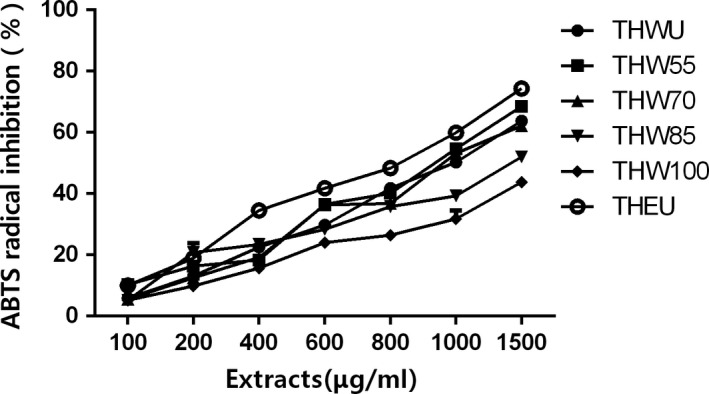
Scavenging ABTS capacity of *Tetrastigma hemsleyanum*

**Figure 4:**
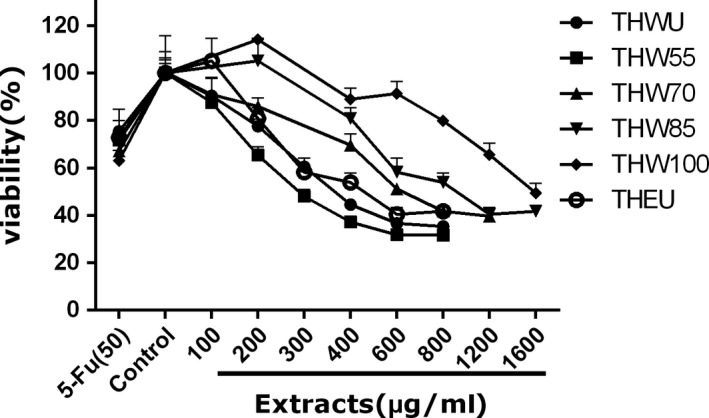
The effect of extracts of different extraction methods on the cell viability of Caco‐2 cells

**Figure 5:**
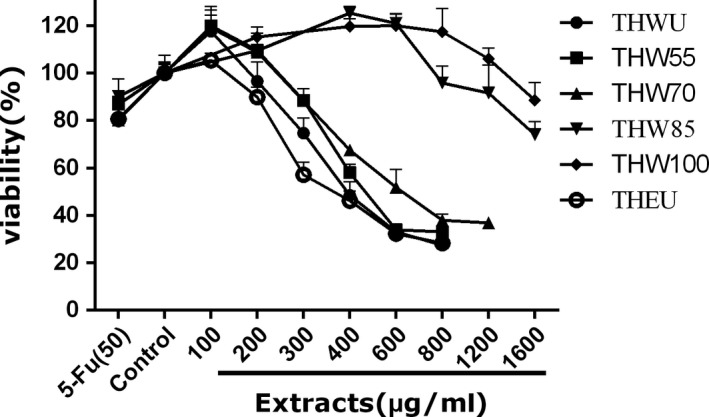
The effect of extracts of different extraction methods on the cell viability of Hela cells

**Figure 6:**
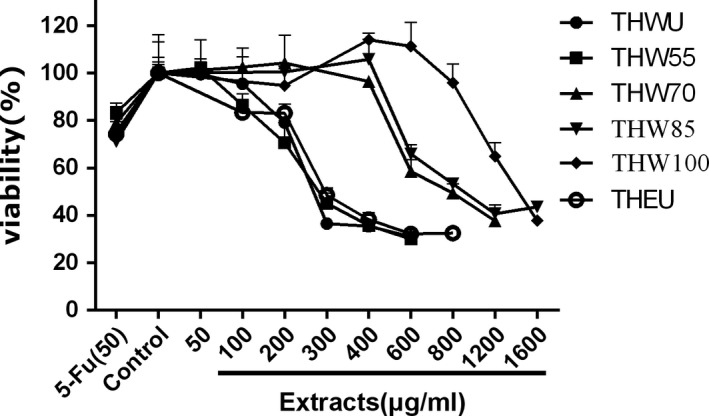
The effect of extracts of different extraction methods on the cell viability of HepG2 cells

**Figure 7:**
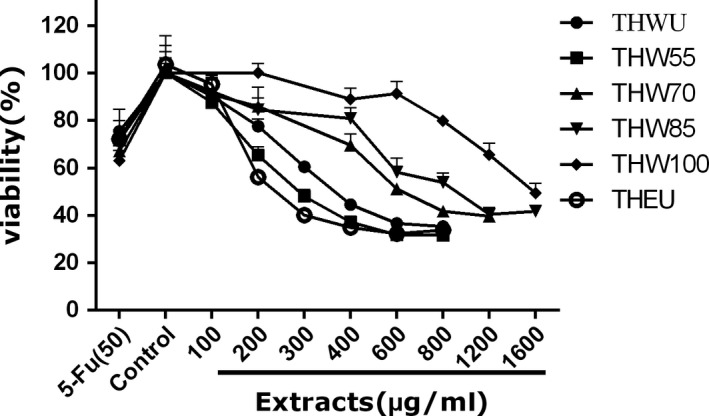
The effect of extracts of different extraction methods on the cell viability of PC12 cells

To determine the effect of extracts on the viability of cancer cells, the viability of human liver carcinoma HepG2 cells, human cervical carcinoma Hela cells, human colon carcinoma Caco‐2 cells, and adrenal pheochromocytoma PC12 cells were observed after extract treatment. 5‐Fluorouracil (5‐FU), a broad‐spectrum anticancer drug, which is widely used in antitumor field, was used as positive control. The MTT assay data of four kinds of cells are shown in Figures 3, 4, 5, 6, 7 and supplementary data (statistical significance was evaluated by one‐way analysis of significance (ANOVA) followed by the Tukey test, and *p* < .05 was regarded to be significantly different in statistics).

Caco‐2 cell is a kind of colon carcinoma cell. As Figure 4 shows, all of the extracts can inhibit the proliferation of Caco‐2 cell. In the tested dose, the cell viability decreased with the increasing dose of the extracts among all extraction methods, while at the same dose of 200, 400, and 600 μg/ml, the cell viability affected by THEU was similar to water extracts of THWU (*p* < .05). However, at the dose of 600 μg/ml, the highest cell viability of 91.4% was observed under THW100 treatment (*p* < .05) which indicated that the bioactivity of extracts decreased with the increasing temperature.

Hela cell is a kind of cervical cancer cell. As Figure 5 shows, under treatment by all the extracts, the cell viability differed from the control group. At the same dose, the cell viability treated by THEU was lower than that treated by the water extracts. Meanwhile, the water extracts of THW55 showed larger cytotoxic than THW100, as the cell viability increased from 55 to 100°C. Especially at the dose of 400, 600, and 800 μg/ml, the cell viability treated by THW55 and THW70 is much lower than THW85 and THW100 (*p* < .05).

HepG2 cell is a kind of cervical carcinoma cell. Figure 6 indicates that the cell viability treated with ethanol extracts decreased with the increasing dose. The cell viability treated with THWU and THW55 both decreased as the dose increased, and the cell viability data showed that there was no significant difference (*p* < .05) between the two extraction methods at the dose of 200 μg/ml. However, the cell viability treated with THW70 and THW85 had the same trends. At the dose of 200 μg/ml, the extracts had little effect on the cell viability (*p* < .05), but at the dose of 400 and 600 μg/ml, the cell viability both decreased. The same phenomenon was observed in THW100.

PC12 cell is a kind of adrenal gland carcinoma cell. The cell viability affected by extracts is shown in Figure 7. The cell viability treated by THEU was lower than water extracts at all tested dose and similar to THW55. The cell viability treated by THWU had the same trends with THW55, and no significant difference was detected at the dose of 100, 200, 400, 600, and 800 μg/ml (*p* < .05). As to the water extracts of THW70, THW85, and THW100, the cell viability decreased with the increasing dose. At the same dose of 600 and 800 μg/ml, the cell viability increased with the increasing temperature (*p* < .05).

To sum up, in the MTT assays of the Hela, HepG2, Caco‐2, and PC12 cells, the extracts can inhibit the cancer cell proliferation. In the tested dose, the cell viability showed dose‐independent manner with extracts. Among all of the extracts, THEU, THWU, and THW55 showed better anticancer activity than THW70, THW85, and THW100. This phenomenon may account for that there is more flavone and polyphenol in THEU, THWU, and THW55 compared with THW70, THW85, and THW100. Several studies have confirmed that flavone extracted from many natural products has antiproliferative activity. In research of Sun, 5 flavonoids including catechin, kaempferol‐3‐rutinoside, rutin, isoquercitrin, and astragalin extracted from *T. hemsleyanum* might be the major antiproliferative compounds (Sun et al., 2015). In the view of Yan, β‐sitosterol as a compound identified from the extracts of *T. hemsleyanum* may have potential in treating cancers (Xiong et al., 2015)*.* The data of Xia suggested that isoquercitrin extracted from *T. hemsleyanum* can inhibit hepatocyte growth factor/scatter factor‐induced tumor cell migration and invasion. Lin (Lin et al., 2016) hold the opinion that resveratrol and kaempferol had antiproliferative activity against MDA‐MB‐435S cell line. In the present study, the synergistic effect of flavonoids, polyphenols, and phytosterol compounds may contribute to the antiproliferative activity on Hela, HepG2, Caco‐2, and PC12 cells, but the key compound in *T. hemsleyanum* playing the role of antiproliferative activity should be determined in further study.

### 3.6 Anti‐inflammatory activity

**Figure 8:**
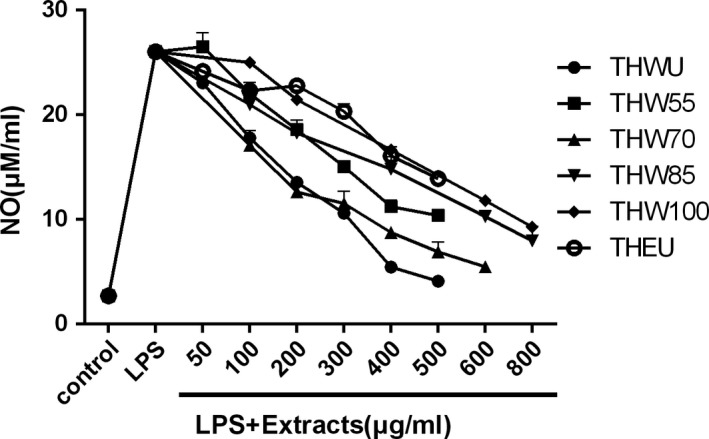
The effect of different extracts on NO production in LPS‐stimulated macrophage cell

To confirm the extracts of *T. hemsleyanum* were not cytotoxic, a cell viability assay was performed. THEU, THWU, and THW55 did not affect the macrophage cell viability at the tested concentration range of 50–600 μg/ml (*p* < .05, data were not shown). Meanwhile, THW70, THW85, and THW100 did not affect the macrophage cell viability at the tested concentration range of 50–800 μg/ml (*p* < .05, data were not shown). The effect of extracts on NO production in LPS‐stimulated macrophage cell is shown in Figure 8 and Table 4. A significant increase of NO production from 2.7 to 27 μM/ml was observed after LPS treatment in Figure 8 (*p* < .05). However, all the extracts can reduce the NO production produced by LPS‐stimulated macrophage. After extract treatment, the NO production of LPS‐stimulated macrophage decreased with the increasing dose. At the same dose of 100, 200, and 400 μg/ml, THWU and THW70 had similar effects on the NO production inhibition and the effects on the NO production inhibition of THWU and TGW70 were better than that of THW55, THW85, THW100, and THEU.

**Table 4:** The effect of different extracts on NO production in LPS‐stimulated macrophage cell

Dose (μg/mL)	NO production (μM/mL)					
	THEU	THWU	THW55	THW70	THW85	THW100
Control	2.7 ± 0.2Ag	2.7 ± 0.2Ah	2.7 ± 0.2Af	2.7 ± 0.2Af	2.7 ± 0.2Ah	2.7 ± 0.2Ah
LPS	26.0 ± 0.4Aa	26.0 ± 0.4A	26.0 ± 0.4Aa	26.0 ± 0.4Aa	26.0 ± 0.4Aa	26.0 ± 0.4Aa
50	24.2 ± 0.2Bb	23.1 ± 0.6Bb	26.5 ± 1.4Aa			
100	22.3 ± 0.3Bc	17.8 ± 0.7Dc	22 ± 1.0Bb	17.1 ± 0.3Db	21.0 ± 0.2Cb	25.0 ± 0.6Ab
200	22.8 ± 0.3Ac	13.6 ± 0.5Dd	18.6 ± 0.9Cc	12.6 ± 0.2Ec	18.3 ± 0.2Cc	21.5 ± 0.5Bc
300	20.3 ± 0.7Ad	10.6 ± 0.3Ce	15.1 ± 0.2Bd	11.5 ± 1.2Ccd		
400	16.1 ± 0.9Ae	5.5 ± 0.2Ef	11.2 ± 0.6Ce	8.8 ± 0.3Dd	14.8 ± 1.2Bd	16.7 ± 0.3Ad
500	13.9 ± 0.2Af	4.6 ± 0.7Bg	10.4 ± 0.4ABe	6.9 ± 0.9Bd		
600				5.5 ± 0.3Ce	10.3 ± 0.2Be	11.8 ± 0.1Ae
800					8.0 ± 1.4Bf	9.3 ± 0.2Af

Note

The difference among different group was compared at *p* < .05 probability level. The capital represents the difference among the same row (different extracts at the same dose), while the minuscule represents the difference among the same line (different dose of the same extracts).

In summary, all of the extracts of *T. hemsleyanum* can reduce the NO production of LPS‐stimulated macrophage. In agreement with our results, alkaloids isolated of *T. hemsleyanum* also showed potent inhibitory activity against NO production in RAW264.7 cells triggered by LPS (Wang et al., 2018). Purified polysaccharide extracted from *Apios americana* Medikus tuber suppressed the release of NO and inflammatory cytokines from LPS‐induced RAW 264.7 cells (Chu et al., 2019). *Ginkgo biloba* sarcotesta polysaccharide has been reported that it could inhibit the secretion of NO in LPS‐stimulated RAW264.7 macrophages (Ye et al., 2018). What's more, similar results were observed in polysaccharide from *Dictyophora indusiate* (Wang et al., 2019), Cycloartane triterpenoids from *Actaea vaginata* (Fang et al., 2019), narciclasine from *Lycoris radiata* (L'Her.) Herb (Shen, Xu, Yang, & Jiang, 2019), and Trichosanhemiketal A and B from the root of Trichosanthes kirilowii Maxim (Ha et al., 2019). However, unlike the results of antiproliferative activity, at the dose of 200 and 400 μg/ml, water extracts of THW55 and THW70 showed better anti‐inflammatory activity than THEU (*p* < .05). This phenomenon indicated that the major decisive compound in *T. hemsleyanum* differs between antiproliferative and anti‐inflammatory activities.

## 4 CONCLUSION

The effect of extracting temperature and solvent on the yield, TFC, TPC, antiproliferative, and anti‐inflammatory activities of *T. hemsleyanum* were evaluated. The results suggested TFC and TPC of water extracts decreased with the increasing extracting temperature and the lowest TFC and TPC were obtained at THW100 with the value of 216.0 ± 4.0 mg rutin/g DW and 90.6 ± 2.9 mg gallic acid/g DW. The EC_50_ of DPPH (504.1 ± 3.8 μg/ml) and ABTS (851.4 ± 3.9 μg/ml) of ethanol extracts were lower than water extracts, and the EC_50_ of water extracts increased with extracting temperature (*p* < .05). The FRAP assay indicated that the antioxidant activity of ethanol extracts was 235.3 ± 18.9 mg VC/g DW which were higher than water extracts whose antioxidant activity decreased with the extracting temperature. All of the three assays of DPPH, ABTS, and FRAP indicated that the ethanol extracts had the best antioxidant activity and antioxidant activity of water extracts in vitro decreased with increasing temperature. In the MTT assays of the Hela, HepG2, Caco‐2, PC12 cells, antiproliferative activity of THEU and THW55 showed dose‐dependent manner to some extent. However, antiproliferative activity of THW85, THW100 doesn't increased with the increaing dose of extractsin the MTT assays of Hela and HepG2 cells. Moreover, THEU and THW55 showed better antiproliferative activity than THW70, THW85, and THW100. The anti‐inflammatory assay indicated that all of the extracts of *T. hemsleyanum* can reduce the NO production of LPS‐stimulated macrophage. The effects on the NO production inhibition of THWU and TGW70 were better than that of THW55, THW85, THW100, and THEU.

On the whole, in view of TFC, TPC, antiproliferative, and antioxidant activities, the ethanol extracts and water extracts of *T. hemsleyanum* obtained at 55°C indicated better bioactivity, while THW70 showed the best anti‐inflammatory activities among all extracts. However, in Chinese herbal medicine processing, oral administration of *T. hemsleyanum* by boiling method is widely used. Though our results suggested that the ethanol extracts and water extracts extracted at 55°C showed potential curative effect, further research is needed to explore about the new processing method of *T. hemsleyanum.* What's more, the major compound in *T. hemsleyanum* play the decisive role of its bioactivities should be determined in further study.

## CONFLICT OF INTEREST

The authors declare that they do not have any conflict of interest.

## ETHICAL APPROVAL

This research does not include any human and animal testing.

## INFORMED CONSENT

Written informed consent was obtained from all study participants.
